# A retrospective analysis of phosphatase catalytic subunit gene variants in patients with rare disorders identifies novel candidate neurodevelopmental disease genes

**DOI:** 10.3389/fcell.2023.1107930

**Published:** 2023-03-28

**Authors:** Ekaterina Lyulcheva-Bennett, Genomics England Research Consortium, Daimark Bennett

**Affiliations:** ^1^ Liverpool Centre for Genomic Medicine, Liverpool Women’s Hospital, Liverpool, United Kingdom; ^2^ Division of Developmental Biology and Medicine, School of Medical Sciences, Faculty of Biology, Medicine and Health, University of Manchester, Manchester, United Kingdom

**Keywords:** phosphatase, phosphatome, disease genes, *de novo* mutation, genome sequencing, rare disorders, mendelian disease, developmental disorder

## Abstract

Rare genetic disorders represent some of the most severe and life-limiting conditions that constitute a considerable burden on global healthcare systems and societies. Most individuals affected by rare disorders remain undiagnosed, highlighting the unmet need for improved disease gene discovery and novel variant interpretation. Aberrant (de) phosphorylation can have profound pathological consequences underpinning many disease processes. Numerous phosphatases and associated proteins have been identified as disease genes, with many more likely to have gone undiscovered thus far. To begin to address these issues, we have performed a systematic survey of *de novo* variants amongst 189 genes encoding phosphatase catalytic subunits found in rare disease patients recruited to the 100,000 Genomes Project (100 kGP), the largest national sequencing project of its kind in the United Kingdom. We found that 49% of phosphatases were found to carry *de novo* mutation(s) in this cohort. Only 25% of these phosphatases have been previously linked to genetic disorders. A gene-to-patient approach matching variants to phenotypic data identified 9 novel candidate rare-disease genes: PTPRD, PTPRG, PTPRT, PTPRU, PTPRZ1, MTMR3, GAK, TPTE2, PTPN18. As the number of patients undergoing whole genome sequencing increases and information sharing improves, we anticipate that reiterative analysis of genomic and phenotypic data will continue to identify candidate phosphatase disease genes for functional validation. This is the first step towards delineating the aetiology of rare genetic disorders associated with altered phosphatase function, leading to new biological insights and improved clinical outcomes for the affected individuals and their families.

## 1 Introduction

The addition or removal of phosphate from proteins is a key regulatory mechanism controlling the activity of most signalling pathways and transcriptional networks that together instruct developmental processes from cell division, programmed cell death, guided cell migration and cell fate determination, to axis formation, tissue patterning and morphogenesis. The regulation of reversible protein phosphorylation by protein kinases and phosphatases is highly conserved (Manning et al., 2002; Chen et al., 2017), and studies in model organisms from flies to mice over the past few decades have elucidated the essential requirement for these enzymes in different cell, tissue and developmental contexts. Clinical investigations of rare conditions, often defined by a prevalence of ≤ 5 cases per 10,000 individuals (Nguengang Wakap et al., 2020), have also begun to reveal the impact of disrupting kinase and phosphatase function during normal human development. However, while phosphatases represent promising candidate genes for a range of rare diseases, to date, no systematic phosphatase-focused analysis of genomic data has been carried out to better understand the association of rare genetic disorders associated with altered phosphatase function. Here, our focus is on the catalytic subunits due to their direct involvement in the enzymatic reactions and because they are structurally and evolutionarily well characterised (Chen et al., 2017).

Rare diseases are often severe, chronic and life-limiting in nature. Despite being individually uncommon, there is an estimated 10,000 rare conditions (Haendel et al., 2020) that collectively affect around 8% of the world population (Posey et al., 2019; Nguengang Wakap et al., 2020). It is widely accepted that approximately 80% of these are genetic in nature. Whilst there is a growing appreciation of the scale and impact of rare disorders, there are predicted to be twice as many novel monogenic conditions yet to be discovered as there are known conditions (Bamshad et al., 2019). Furthermore, genotype-phenotype correlations are poorly understood for many genes and the full spectrum of disease-causing variants in recognised disease genes has not yet been fully elucidated. Consequently, most individuals affected by rare disorders remain without a molecular diagnosis for their conditions, even after extensive genetic testing (Taylor et al., 2015; Retterer et al., 2016; Boycott et al., 2017; Splinter et al., 2018; Smedley et al., 2021). This has a large impact on affected individuals and family members because a molecular diagnosis is the pre-requisite for prognosis, appropriate screening and/or treatment, predictive testing for relatives, and facilitating reproductive choices. Consequently, there is an unmet need to improve diagnostic yield in rare inherited diseases and improve the experience and outcomes for affected individuals and their at-risk relatives.

Recent advances in genome sequencing have revolutionised rare disease gene discovery and diagnosis (Bamshad et al., 2019). Traditionally, clinical genetics practice relied on a phenotype-centred approach, where genetic testing represented the last step in a prolonged diagnostic process for selected gene targets in a minority of patients. Subsequently, genome/exome sequencing initiatives have aimed to improve diagnostic yield and outcomes for patients with rare disorders, as well as augment novel disease gene discovery to create opportunities for scientific and medical innovation (Wright et al., 2015; Bamshad et al., 2019; Turro et al., 2020). The 100 kGP has been the largest national sequencing project so far, which aimed to sequence 100,000 genomes from 85,000 individuals within the United Kingdom health service between 2015 and 2018 (Smedley et al., 2021). Such data has vast, albeit not yet fully realised, diagnostic potential, and has revealed a myriad of novel genetic variants, including variants in phosphatase genes that have not previously been linked to rare developmental disorders.

The diagnostic yield of the 100 kGP so far has been estimated to be between 22% and 35% (Turnbull et al., 2018; Smedley et al., 2021). Current limitations of diagnostic genome and exome sequencing is that sequence analysis is restricted to pre-defined panels of known disease genes that are selected according to patients’ phenotype. Such panels reflect the current understanding of phenotype-genotype correlations and, consequently, relevant genes may not be analysed because there is insufficient evidence linking them to a given disorder at the time of testing. It is estimated that 250 new disease genes are reported annually (Seaby et al., 2021), but this knowledge is not automatically applied to existing genomic data. Clearly, in view of the ever-growing scientific understanding of rare disease aetiology, systematic re-analysis of genomic data is necessary to improve diagnostic yield and clinical outcomes (Wright et al., 2018). This remains an ambitious undertaking and various strategies have been proposed to uplift gene discovery in rare diseases (Seaby et al., 2021). Gene-centred approaches have shown promise, and these may be further refined by focusing on defined gene groups of interest (Best et al., 2022; Seaby et al., 2022). Here we present a systematic survey of genomic and phenotypic data from rare disease trios in the 100 kGP to identify potential disease-causing variants in genes encoding phosphatase catalytic subunits. All trios included in this study were recruited to the 100 kGP due to an unmet diagnostic need following standard assessment and genetic testing. We utilise a gene-to-patient approach to match *de novo* variants in phosphatase genes encoding catalytic subunits to phenotype data recorded as discrete Human Phenotype Ontology (HPO) terms.

## 2 Methods

### 2.1 Genes under investigation

A list of 189 phosphatases, their phylogenetic classification, predicted substrate type and other descriptors were taken from (Chen et al., 2017), see (Supplementary Table S1). This included catalytic subunits from the following families: AP, alkaline phosphatase (4 genes), DSP, dual specificity phosphatase (40 genes) Myotubularin (15 genes), PTEN, phosphatase and tensin homolog (8 genes), PTP, protein tyrosine phosphatase (37 genes), Sac, Sac domain-containing phosphatase (5 genes), CDC25, cell division cycle 25 (3 genes), EYA, eyes absent family (4 genes), FCP, RNA polymerase II carboxy-terminal domain phosphatase (8 genes), NagD, NagD family of HAD-fold phosphatases (5 genes), HP1, histidine phosphatase 1 (12 genes) HP2, histidine phosphatase 2 (8 genes), PPM, PPM/PP2C family (20 genes), PPP (13 genes) and PPP-like phosphatases (2 genes), RTR1 (1 gene), other (3 genes).

### 2.2 100 kGP dataset

Data was obtained *via* the secure Genomics England (GEL) research environment following information governance training as a members of the Genomics England Clinical Interpretation Partnership (GECIP): Enhanced Interpretation Domain, with approved project ID: 720—Towards understanding the aetiology of rare genetic disorders associated with altered phosphatase function. This provided access to data for 34,082 probands from 35,002 families (October 2022) analysed using the Illumina Starling pipeline and passing quality control parameters (Smedley et al., 2021). Patients were recruited to the study based on a residual unmet diagnostic need with a presumed underlying rare genetic disorder following standard clinical assessment and genetic investigations. These rare conditions were enriched in neurodevelopmental disorders, but also included disorders of growth, metabolism, the renal tract, skeletal and cardiovascular systems, vision, dysmorphic and congenital anomaly syndromes, tumour syndromes, and haematological and immunological disorders. After variant calling, filters were routinely applied to remove common variants, variants with no predicted impact and those that did not segregate with disease. Phenotypic descriptors, as assigned by the referring clinician, were recorded using Human Phenotype Ontology (HPO) terms. Variant details, associated patient phenotypic information and Genomic Medicine Centre (GMC) outcomes were extracted using the R LabKey package.

### 2.3 Additional databases and genome analysis tools

We used MARRVEL (Model organism Aggregated Resources for Rare Variant ExpLoration) *via* http://marrvel.org/human/batch/genes, to extract data from a range of human databases and assist in variant prioritisation (Wang et al., 2017). Additional information on existing syndromes was obtained from OMIM (https://omim.org/) and *via* literature searches. LOEUF (loss-of-function observed/expected upper-bound fraction) metrics were also compared across other predictive measures of intolerance to loss-of-function obtained from DECIPHER (https://www.deciphergenomics.org/). The predicted effect of genomic variants on known transcripts was determined using the Ensembl Variant Effect Predictor http://www.ensembl.org/Homo_sapiens/Tools/VEP (McLaren et al., 2010), with liftover of genome coordinates and annotation from GRCh37 to GRCh38 genome assembly using the UCSC LiftOver tool http://genome.ucsc.edu/cgi-bin/hgLiftOver, where necessary. Estimates of variant frequency in the general population were obtained from the genome aggregation database - gnomAD v3.1.2 or v2.2.1 (https://gnomad.broadinstitute.org/). To assist in the validation of candidate novel disease genes, additional patient cases for selected genes were identified from DECIPHER (Database of Genomic Variation and Phenotype in Humans Using Ensembl Resources) (https://www.deciphergenomics.org/) (Foreman et al., 2022). We curated high-level HPO terms for 100 kGP patients to enable comparison of patient phenotypes for selected variants across 100 kGP and DECIPHER. For example, “intellectual disability” was upscaled to “abnormality of the nervous system” (e.g. see https://hpo.jax.org/app/browse/term/HP:0000707).

### 2.4 CADD scores

To assess potential impact of variants identified for candidate disease genes we analysed Combined Annotation-Dependent Depletion (CADD) scores, which integrate diverse annotations for each variant, including allelic diversity, pathogenicity and annotations of functionality, into a single measure (Kircher et al., 2014; Rentzsch et al., 2021; Rentzsch et al., 2019). CADD scores were obtained by searching precomputed PHRED-like (-10*log10 (rank/total)) scaled C-scores for all possible human single-nucleotide variants (8.6 × 10^9^) *via* https://cadd.gs.washington.edu/snv.

## 3 Results

### 3.1 Frequency of variants amongst different protein phosphatase families

**FIGURE 1:**
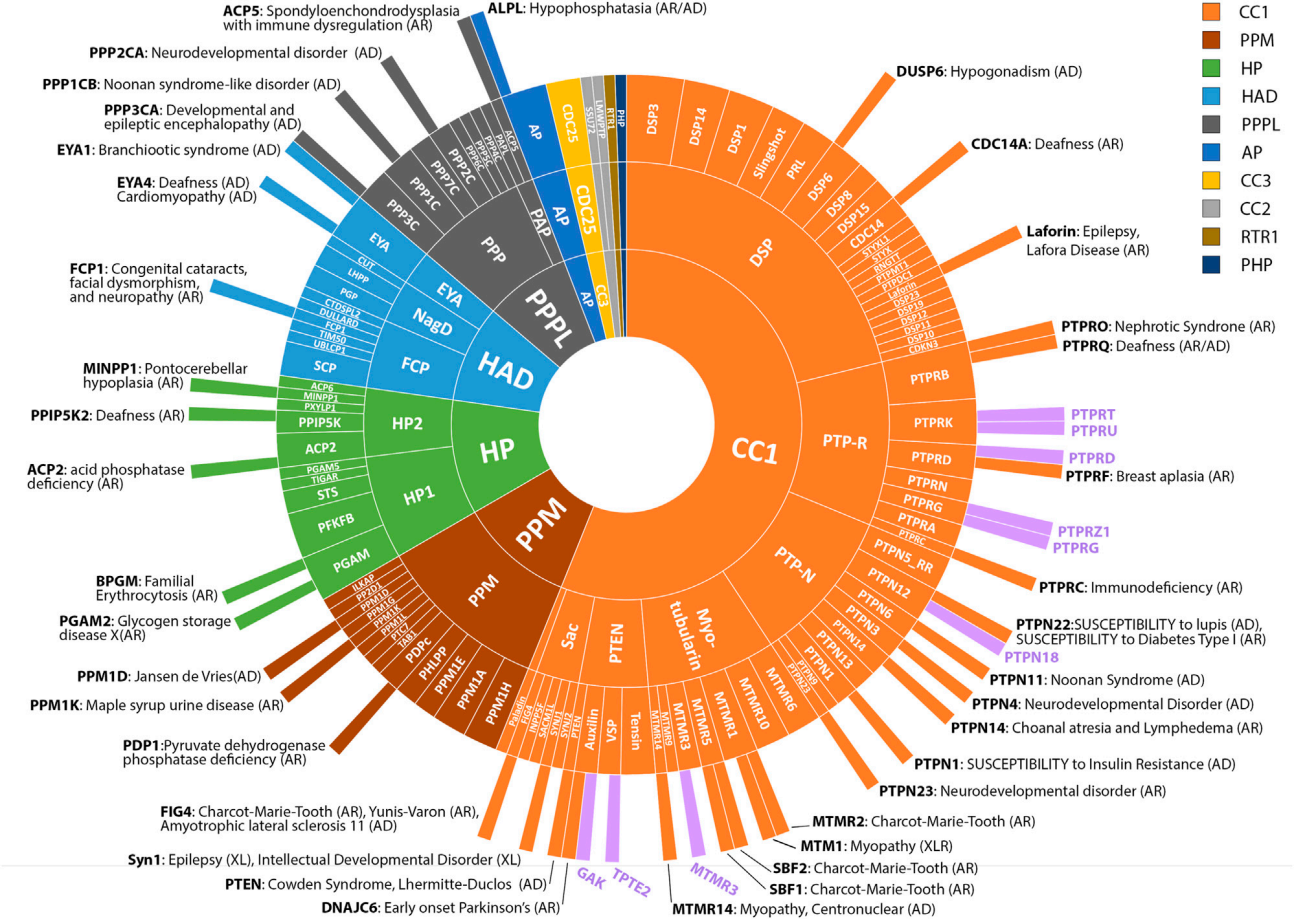
Known and putative disease genes amongst the Phosphatome. Radial plot showing grouping of 189 phosphatases into folds, families and subfamilies (outwards from inner circle) according to (Chen et al., 2017). The majority of protein phosphatases belong to the CC1 class (106 genes) and share a common structural fold and common catalytic CX5R motif. Other major classes include: PPM (20), HP (20), HAD (17), PPPL (15), AP (4), CC3 (3), CC2 (2), RTR1 (1) and PHP (1). See key for colour coding. The outermost circle depicts disease genes reported in OMIM or the literature as of October 2022. Inheritance pattern of associated conditions is indicated (AD, autosomal dominant; AR, autosomal recessive; XLR, X-linked recessive). Novel candidate disease genes reported in this study are shown with purple bars.

**FIGURE 2:**
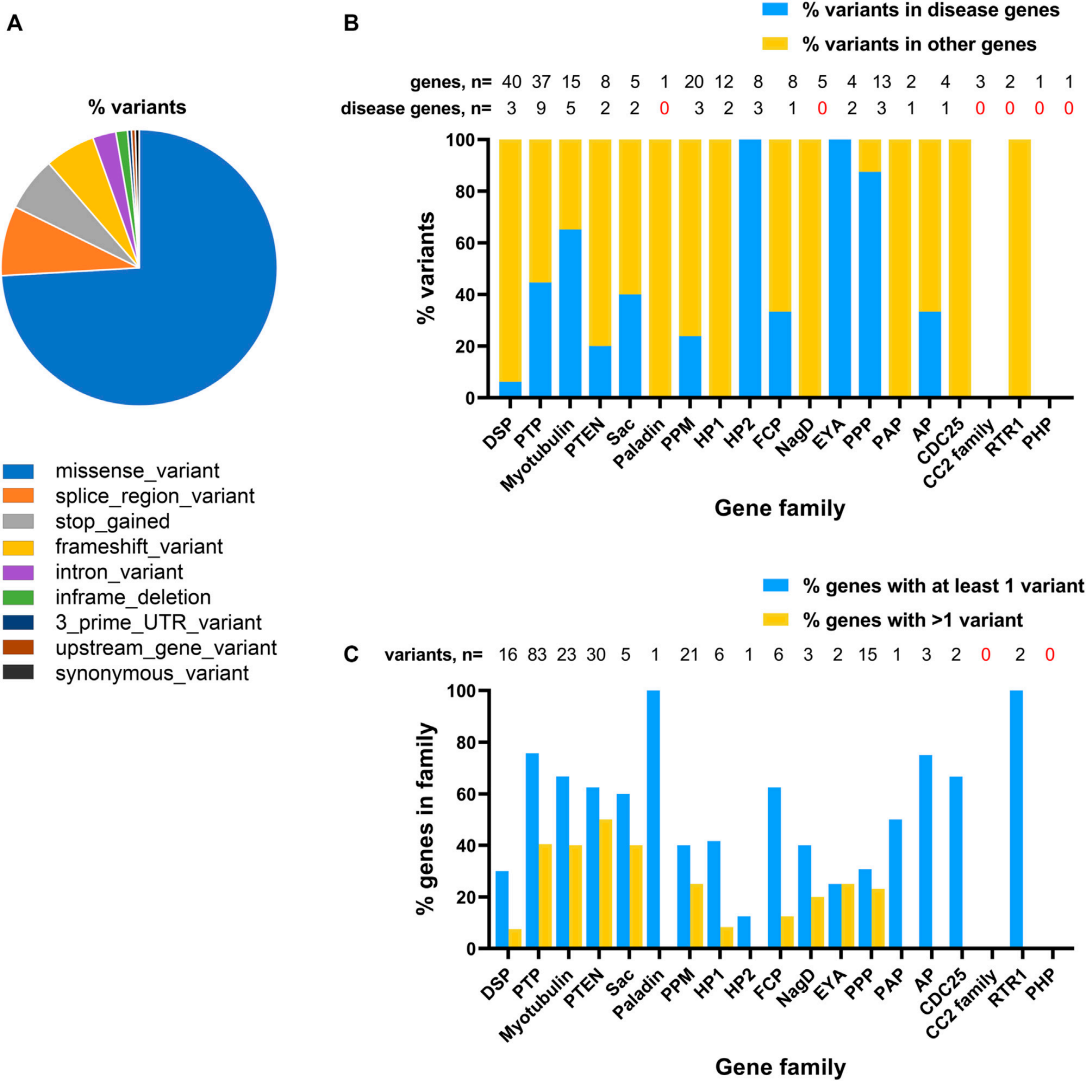
Distribution of *de novo* variants for phosphatase genes identified amongst probands recruited to the 100,000 Genome Project. **(A)**, Predicted consequences of phosphatase catalytic subunit variants on MANE-select transcripts. **(B)**, Plot of relative abundance of variants in known disease genes (blue bars) and other phosphatase genes (yellow bars) for each phosphatase family. Shown above the graph is the number of genes and number of disease genes in each family. **(C)**, Plot showing proportion of genes in each of the different phosphatase families harbouring at least one variant (blue bars) or more than one variant (yellow bars) in affected probands. Total number of variants identified in each family is shown above the graph.

Given the broad role of phosphatases in development and the availability of genomic data for undiagnosed rare disease patients, we considered it timely to systematically assess frequency of genetic variants in phosphatase genes and their possible association with rare-developmental disorders. We focused our attention on *de novo* changes identified in a cohort of patients recruited to the 100 kGP. We studied 189 genes encoding the human phosphatome (Figure 1), as defined by a recent genome-wide, cross-species survey of phosphatase catalytic subunit genes, which can be classified according to their structural folds and membership of subfamilies based on evolutionary origins (Chen et al., 2017). Amongst patients enrolled into the 100,000 Genomes Project (as of October 2022) we determined there were 220 rare *de novo* variants in 92 phosphatase genes (49% of the phosphatome), including 39% novel variants that are not present in the Ensembl database (see Supplementary Tables S1 and S2). The majority of MANE-select variants (Morales et al., 2022) were determined to be missense mutations (74%), with splice region variants (8%), premature stop (6%) and frameshift mutations (6%) being prevalent among the other classes (Figure 2A; Supplementary Table S2).

22 out of 37 phosphatase genes known to be associated with a rare disorder (as recorded in OMIM as of October 2022) were represented by at least one variant (60%). The most highly represented genes of this type were: PTPN11 (24 variants); PPP1CB (8 variants), PTEN (6 variants). Although most patients carrying variants in these genes had received a diagnosis, one pathogenic variant in each of PTPN11, PPP1CB and PTEN had not been reported as a diagnostic outcome. This is most likely because the recorded phenotypes did not prompt analysis of these genes, reinforcing the need for reinterrogation of the available genomic data in undiagnosed 100 kGP patients.

Although many of the variants we identified mapped to known disease genes, 61% of the total number of variants resided in genes that had not been previously associated with rare conditions, revealing a large potential for novel disease gene discovery (Figure 2B). The coverage of variants across different subfamilies of phosphatases varied considerably. Among multigene phosphatase groupings (i.e., not including single genes, such as Paladin), the maximum coverage of variants was in the PTP family (∼75% of genes/family with variants), the minimum coverage was in the CC2 enzymes (no variants), Figure 2C and Supplementary Table S1.

### 3.2 Identifying candidate novel disease genes

In total, we detected *de novo* variants in 70 genes not previously associated with a rare developmental disorder. To identify novel disease gene contenders we considered focusing on genes predicted to be intolerant to gene inactivation. This principle can be used to prioritise genes that have fewer deleterious loss-of-function (LoF) variants in the general population than would be expected by chance (Seaby et al., 2022) because natural selection removes variants that reduce survival and ability to reproduce (Karczewski et al., 2020). One metric to describe this is the loss-of-function observed/expected (O/E) upper-bound fraction (LOEUF). Genes in the first decile of LOEUF, with a score < 0.2, are amongst the most enriched for OMIM haploinsufficient disease genes (Karczewski et al., 2020). However, a survey of autosomal dominant inherited phosphatase disease genes in our dataset reveals that only three out of 13 (23%) disease genes (PTPN11, PPP1CB and PPP3CA) would meet this LOEUF criterion, with seven genes scoring 0.26–0.69 and three genes (PPM1D, PTPN22, PTPRQ) obtaining LOEUF scores of >1.0. Therefore, we adopted a filtering strategy that focused primarily on identifying genes with variants associated with matching phenotypes in unrelated patients.

The classification criteria and number of genes assigned to each of the categories are shown in Table 1. Class 1 genes included matches to patients in recent published datasets (after the end of 100 kGP patient recruitment, post 2018). Class 2 and 3 genes were identified as having variants in two or more unrelated kindreds in GEL with the same phenotype. We distinguished Class 2 and 3 genes using the upper bound of the O/E confidence interval LOEUF < 0.35 as a threshold. Variants were not considered in our analyses if an alternative diagnosis had been proposed by clinicians as recorded in the GEL research environment (GMC outcomes). This approach identified two class 1 genes: PPP2CA and PTPN4, for which multiple variants have recently been reported as being linked to neurodevelopmental disorders (Reynhout et al., 2019; Chmielewska et al., 2021), validating our approach. Pathogenic mutations in PPP2CA have been reported across the coding region of the gene (Reynhout et al., 2019). We uncovered missense mutations in exon two and three that add to spectrum of likely deleterious PPP2CA mutations associated with neurodevelopmental disorder and intellectual disability.

**TABLE 1:** Criteria for classification of putative novel phosphatase disease genes. Rules used to assign genes to class 1–4 are shown together with the number of phosphatase genes belonging to each class. Known disease genes as reported in OMIM or in the literature prior to 2019 are not included.

Gene class	Classification rule	Number
Class 1	Identified as candidate disease gene in the literature after 2019; at least one variant identified in GEL with at least 1 HPO term matching between affected individuals and reported cases	2
Class 2	Variants with LOEUF <0.35 identified in 2 or more unrelated kindred in GEL with at least 1 HPO term matching between affected individuals	5
Class 3	Variants not meeting LOEUF threshold, identified in 2 or more unrelated kindred in GEL, with at least 1 HPO term matching between affected individuals	4
Class 4	Variants not meeting LOEUF threshold, with no matching HPO terms between affected individuals; or, no variants available	123

### 3.3 Assigning confidence in novel candidate disease genes

Having established that our approach was capable of identifying bone fide rare-disease genes, we focussed our attention on novel genes. After excluding variants in known disease genes, analysis of 134 variants using our selection criteria (Table 1) uncovered nine Class 2 and 3 genes (PTPRD, PTPRG, PTPRT, PTPRU, PTPRZ1, MTMR3, GAK, TPTE2, PTPN18) representing candidate rare developmental disorder genes (Table 2). Analysis of family structures for affected probands confirmed that all cases contained full parent offspring trios or families with more than three participants. Diagnostic yield amongst such families is approximately twice that for singletons for whom no other family member was recruited for whole genome sequencing (Smedley et al., 2021). To prioritise genes for further analysis we analysed variant abundance in the general population and potential pathogenicity. 67% of variants were not present in the general population database, consistent with them being deleterious changes. The highest variant frequency was 1.6 × 10^−4^. We assessed potential pathogenicity of variants using CADD scores, which rank variants relative to all possible substitutions of the human genome (8.6 × 10^9^). The median score for 100 kGP variants we identified (Table 1) is 25.1, placing them in the top 1% most deleterious possible substitutions.

**TABLE 2:** Candidate novel phosphatase disease genes. Gene class is defined as in Table 1. Confidence score was derived by allocating points as follows: LOEUF < 0.35 (1 point), two variants CADD >20 (2 points) or, one variant CADD >20 and one variant CADD>10 (1 point), ≥1 DECIPHER or published variant (1 point); >1 matching HPO term (1 point). See text for details of LOEUF and CADD scores. Max Freq, maximum allele frequency in gnomadAD v2.1.1. HPO terms are as follows: ADHD (HP:0007018), Abnormality of the nose (HP:0000366); Autistic behavior (HP:0000729); Delayed fine motor development (HP:0010862); Delayed gross motor development (HP:0002194); Delayed speech and language development (HP:0000750); Global developmental delay (HP:0001263); Generalized hypotonia (HP:0001290); Hyperacusis (HP:0010780); Intellectual disability (HP:0001249); Joint hypermobility (HP:0001382); Macrocephaly (HP:000256); Microcephaly (HP:0000252); Morphological abnormality of the CNS (HP:0002011). Elevated HPO terms used for Decipher patients: Abnormality of the nervous system (HP:0000707); Growth abnormality (HP:0001507).

Class and confidence	Gene and LOEUF score	Consequence and pathogenicity (CADD) score for 100 KGP variants		Max Freq	Shared HPO Terms across at least 2 patients in GEL (*or with report in recent publication)	DECIPHER variants (with shared upscaled HPO term) and CADD score		Max Freq.
Class 1 Conf. 5 (High) Ref. (Reynhout et al., 2019)	**PPP2CA** (0.26)	• Missense• Missense	25.5 27.0	None None	Microcephaly; Delayed speech and language development; Intellectual disability; Global developmental delay; Morphological abnormality of the CNS; Delayed gross motor development; *Also overlap with Ref. (Reynhout et al., 2019)	None	N/A	N/A
Class 2 Conf. 5 (High)	**PTPRD** (0.11)	• Missense • Missense	21.4 22.1	None None	Delayed speech and language; Delayed gross motor development	• Missense • Splice acceptor variant (Abnormality of the nervous system)	26.6 29.0	2.0e-5 None
Class 2 Conf. 5 (High)	**PTPRT** (0.18)	• Splice donor variant • Missense	32.0 27.7	None None	Autistic behaviour, Delayed speech and language development, Intellectual disability, Global developmental delay	• Missense • Missense • Missense • Missense (Abnormality of the nervous system)	26.0 31.0 23.5 22.8	None 6.6e-6 2.6e-5 None
Class 2 Conf. 4 (Med)	**MTMR3** (0.26)	• Missense • Missense	26.6 32.0	None None	Delayed speech and language development; Intellectual disability; Global developmental delay; Delayed gross motor development; Delayed fine motor development.	None	N/A	N/A
Class 3 Conf. 4 (Med)	**GAK** (0.41)	• Missense • Missense • Missense	23.8 25.1 22.8	6.6e-6 4.0e-6 1.3e-5	Macrocephaly; Delayed speech and language development; Global developmental delay; Delayed gross motor development	• Frame shift (Abnormality of the nervous system)	N/A	None
Class 3 Conf. 4 (Med)	**TPTE2** (0.94)	• Missense (3 cases) • Stop gain• Missense	20.7 34 15.5	7.1e-5 None None	Delayed speech and language development; Intellectual disability; Global developmental delay; Joint hypermobility; Delayed gross motor development; Delayed fine motor development.	• Splice donor variant (Abnormality of the nervous system)	22.9	1.1e-3
Class 1 Conf. 3 (Med)	**PTPN4** (0.28)	• Missense	24.2	None	*Overlap with refs (Chmielewska et al., 2021; Szczaluba et al., 2018): Macrocephaly; Autistic behaviour; Delayed speech and language development; Hyperacusis; ADHD; Abnormality of the nose.	• Missense • Frameshift (Abnormality of the nervous system)	24.2 N/A	None 6.6e-6
Class 2 Conf. 3 (Med)	**PTPRZ1** (0.29)	• Missense • Missense	26.2 7.8	7.2e-5 1.6e-4	Generalized hypotonia; Intellectual disability	• Missense (Abnormality of the nervous system)	3.2	1.9e-4
Class 2 Conf. 3 (Med)	**PTPRU** (0.32)	• Missense • Missense • Missense	32 26 24	None 6.6e-6 4.6e-5	Intellectual disability	None	N/A	N/A
Class 3 Conf. 3 (Med)	**PTPRG** (0.45)	• Missense (poly T) • Missense	ND 27.2	None None	Global developmental delay	• Stop gained (Abnormality of the nervous system; Growth abnormality)	35.0	
Class 3 Conf. 2 (Low)	**PTPN18** (0.98)	• Splice reg-ion variant • Missense	14.7 10.0	None None	Autistic behaviour; Delayed speech and language development; Intellectual disability	• Frame shift (Abnormality of the nervous system)	N/A	6.6e-5

To provide further evidence that gene perturbation is linked to the associated phenotypes, we interrogated cases reported in the Decipher database (Wright et al., 2015) to find additional *de novo* variants in the selected genes with matching high level HPO terms. Seven out of nine genes had variants associated with matching phenotypes in Decipher. Although there is some potential for patient overlap in Decipher and 100 kGP, all but one variant was distinct between the two databases and therefore come from unrelated patients. To rank the candidate genes for future follow up, we used a points-based system to assign a confidence score in the candidate genes (Table 2). Together, our findings suggest that phosphatases represent promising candidate genes for a range of undiagnosed developmental disorders and in particular, neurodevelopmental syndromes, which are relatively underexplored.

## 4 Discussion

Genome data reanalysis to identify clinically associated phosphatase catalytic subunit variants in the 100 kGP.

We utilised here a gene-centred approach to survey the frequency of clinically-associated variants in phosphatase catalytic subunit genes in the 100 kGP cohort of patients. 100 kGP is enriched in patients with rare neurodevelopmental disorders, offering a unique opportunity to reveal the under-recognised contribution of phosphatases to these conditions. 0.65% of index cases in this cohort were found to harbour at least one phosphatase gene variant. More than 88% of the variants we identified are predicted to alter the protein coding sequence of MANE-select transcripts, providing a potential mechanism of action by which genetic alteration leads to the associated clinical presentations.

We went on to reanalyse genomic data, together with phenotypic information recorded as discrete HPO terms to identify novel candidate phosphatase disease genes not revealed by standard 100 kGP diagnostic protocols. Indeed, this approach is independent of gene panels, which are currently used in patient diagnosis and only include known disease genes, change over time, and depend on careful phenotyping for appropriate selection. Our approach was validated by the identification of a number of well-characterised phosphatase disease genes, which were subsequently filtered out for lack of novelty. Less well-established but recognised disease genes (PPP2CA, PTPN4) were also identified as part of this reanalysis attracting similar confidence scores to novel candidate disease genes. Notably, all of the novel genes we identified belong to the CC1 group of phosphatases, with six belonging to the PTP family of tyrosine phosphatases, two to the PTEN family and one to the Myotubularin family. The CC1 group, possessing 73% of the variants, constitutes 56% of the total number of phosphatases we have analysed, at least partially explaining the enrichment of novel disease genes in these families.

The reason why some highly constrained phosphatases are not represented by variants in the 100 kGP dataset, might be because the study only recruited living patients. Some phosphatases are likely to play essential and pleiotropic roles early in development and pathogenic variants may therefore result in embryonic lethality. For instance, we did not recover any variants in PPP6C, which is a highly constrained gene that is essential for post-implantation embryogenesis (Ogoh et al., 2016).

By focusing on *de novo* variants, any pathogenic variants we identified would be expected to be associated with dominantly inherited conditions. It also follows that since we did not examine biallelic variants, any disorders inherited in a recessive fashion will be under-represented (Posey et al., 2019). This may account for under-representation of some gene families such as the HP1 and HP2 families, which together account for 10% of the phosphatases but possess only 3% of the variants we identified; all of the recognised disease genes in this family thus far are inherited in a recessive manner (see Figure 1).

### 4.1 Non-catalytic phosphatase subunits in rare developmental disorders

We have focused here on phosphatase catalytic subunits. However, it is important to note that the majority of PPP catalytic subunits complex with regulatory subunits that direct these enzymes’ specific roles. Consequently, non-catalytic subunits may also represent a source of promising disease gene candidates. Indeed, nine genes encoding regulatory subunits of PP1 and PP2A are known to be associated with heritable disorders (Vaneynde et al., 2022). However, systematic analysis of the non-catalytic subunits poses a challenge because of the large number of disparate proteins that interact with PPP catalytic subunits. This is particularly true of PP1 which is thought to bind more than 180 PP1-interacting proteins (Verbinnen et al., 2017), with diverse functions ranging from molecular chaperones to signalling molecules and transmembrane receptors and channels. Moreover, because many PPP binding proteins act as multivalent binding or scaffold proteins, non-catalytic subunits often have additional functions unrelated to their phosphatase-binding properties. For example, SARA, Spinophilin and various A-kinase anchoring proteins (AKAPs), bind to a variety of PPP catalytic subunits and exert diverse regulatory functions by controlling the assembly, trafficking and localisation of signalling complexes containing multiple components (Bennett and Alphey, 2002; Wong and Scott, 2004; Shaw and Filbert, 2009; Rozes-Salvador et al., 2018). Additional roles for PPP regulatory proteins that are not exclusively dependent on the phosphatase catalytic activity are continually being uncovered, highlighting the complexity of their functions (Sakaguchi et al., 2022). These features of the non-catalytic subunits complicate the ability to predict phosphatase dependent and independent effects of variants in these genes. In future, careful scrutiny of variants in genes encoding non-catalytic subunits, accompanied by analysis of potential phenotypic overlap with the relevant catalytic subunits will begin to address this issue. The findings that we report here sets the foundation for ongoing efforts in this direction.

### 4.2 PTPRD and PTPRT variants are potentially linked to neurodevelopmental disorders

Foremost of the novel disease gene candidates we have identified is PTPRD, for which there are several lines of evidence supporting its role in neurodevelopmental disorders. *In situ* hybridization analysis has revealed that murine PTPRD is expressed in the specialized regions of the brain including the hippocampal CA2–CA3 region, thalamic reticular nucleus, piriform cortex, olfactory bulb, olivary nucleus and spinal motor neurons (Mizuno et al., 1993; Sommer et al., 1997; Schaapveld et al., 1998; Shishikura et al., 2016). Moreover, studies into the fundamental cell and developmental roles of PTPRD in mice have shown roles for PTPRD in neural precursor and cortical development (Tomita et al., 2020), axonal growth and pathfinding (Uetani et al., 2006). Clinically, genome wide association studies have linked PTPRD variants with several neural disorders, including: 1) rare copy number variation with autism spectrum disorders (ASDs) (Pinto et al., 2010), attention-deficit/hyperactivity disorder (ADHD) (Elia et al., 2010), and obsessive-compulsive disorder (OCD) (Gazzellone et al., 2016); 2) Single nucleotide polymorphisms with schizophrenia (Li et al., 2018); and, 3) non-coding variants associated with restless leg syndrome (Schormair et al., 2008; Yang et al., 2011). Our identification of *de novo* PTPRD protein coding changes in patients affected by motor defects and speech and learning difficulties raise the prospect of there being a neurodevelopmental syndrome associated with altered PTPRD function. Notably, Ptprd knockout mice also show defects in motor development as well as learning and memory (Uetani et al., 2000). This and other models may therefore offer an ideal opportunity to assess functional effects of the variants we have identified.

PTPRT is also a prominent neurodevelopmental disease gene candidate. Expression of PTPRT RNA and protein is enriched in the brain (McAndrew et al., 1998; Uhlen et al., 2015), and in early development PTPRT transcripts are distributed throughout the murine brain and spinal cord (McAndrew et al., 1998), suggesting a role in CNS function. Functional studies in mice indicate that these roles are likely to include hippocampal neurogenesis, synaptogenesis and dendritic arborization of hippocampal neurons (Lim et al., 2009; Lee, 2015; Lim et al., 2020). Notably, an individual with intellectual disability and a *de novo* PTPRT variant has previously been reported (Schuurs-Hoeijmakers et al., 2013). Further cases will help validate the role of PTPRT in neurodevelopmental disease. We are now building case series and undertaking functional analyses to confirm genotype-phenotype relationships for PTPRD, PTPRT and other genes we have identified.

### 4.3 Limitations of available phenotypic information

Our gene discovery approach relies heavily on finding a match between phenotypes of unrelated patients with *de novo* variants in a given gene of interest. However, since the cohort is enriched with similar phenotypes there is a risk of false positives arising. Conversely, due to the variability and non-standardised application of HPO terms used to describe similar phenotypes, there may also be false negatives. Our previous analysis across two genomic medicine centres (North West Coast GMC and Greater Manchester GMC, n = 3,212 index cases) suggests that diagnostic yield is poor in cases with limited or non-specific phenotypic information, reflected in a low recorded number (≤ 15) of HPO terms (E Lyulcheva-Bennett 2020; personal communication). The occurrence of inaccurate or incomplete phenotype records highlights the need to collaborate with clinicians to obtain comprehensive and up-to-date phenotypic data.

### 4.4 Variant frequency in the general population

The majority of novel variants in candidate disease genes that we discovered are not found in the general population database, gnomAD. Those variants that were found at low frequency might be non-pathogenic, or pathogenic with incomplete penetrance, modified by cis-regulatory variation, or associated with adult-onset disease (Gudmundsson et al., 2021). We are unable to distinguish between these possibilities at the current time. We used CADD scores to obtain a preliminary measure of potential pathogenicity. Almost all identified genes have at least one variant with a CADD score >25, which is comparable to well characterised pathogenic lesions in known phosphatase disease genes (e.g., PTPN11-T73I, CADD 25.6; PPP1CB-P49R, CADD 26.7).

### 4.5 Haploinsufficiency metrics in gene-centred approaches to disease gene discovery

Haploinsufficiency metrics have gained popularity in the field of disease gene discovery as they can be used to focus the scope of genomic data reanalysis and to inform disease etiology. We did not use these metrics for candidate disease gene identification because known phosphatase disease genes span a broad spectrum of haploinsufficiency tolerance. In addition, natural selection is blind to phenotypes that do not affect the ability to reproduce. Therefore, genes associated with later-onset phenotypes, or phenotypes not impacting on reproduction, exhibit much weaker intolerance to inactivation and will most likely not be picked up when applying haploinsufficiency constraint-based metrics. Moreover, the relationship between genetic constraint and molecular pathogenesis is likely to be complex. While the pathogenic mechanism of highly constrained genes might be assumed to be loss-of-function, this is not always the case. For instance, pathogenic gain-of-function mutations in the constrained PTPN11 gene (LOEUF 0.14) are a known cause of Noonan’s Syndrome. On the other hand, loss-of-function PTPN11 mutations give rise to an allelic disorder (Noonan’s Syndrome with Multiple Lentigines), with overlapping clinical features (Solman et al., 2022). Similarly, variants in genes that are not predicted to be haploinsufficient, can nevertheless be pathogenic, for instance by acting in a dominant-negative fashion, as is the case for C-terminally truncating mutations in PPM1D (LOEUF 1.1) (Jansen et al., 2017).

### 4.6 From patients to models of developmental disorders

With the aspiration to sequence five million more genomes in the United Kingdom as part of routine clinical practice, and with increasing numbers of patients undergoing whole genome sequencing globally, we anticipate that reiterative gene-centred analysis of abundant genomic and phenotypic data will help to identify further phosphatase gene variants for functional validation. This should go hand-in-hand with clinical gene discovery clinics re-examining unsolved cases. Our current focus is on expanding the scope of our analysis to include *de novo* variants affecting the phosphatase regulatory subunits and uncover novel recessive phosphatase disorders. The reanalysis of genomic data provides the first step towards systematically delineating the aetiology of rare genetic disorders associated with altered phosphatase function. Many of the known developmental disease genes were first identified in model species based on their role in fundamental developmental processes. Consequently, experimental model systems provide a rich resource with which to dissect mode of action, establish disease causality, and determine variant pathogenicity. Improved information sharing among clinicians, genomic scientists and biologists will assist in efforts to aid phosphatase disease gene discovery and delineate genotype-phenotype relationships.

## Genomics England Research Consortium

**Ambrose J. C.**, Genomics England, London, United Kingdom, **Arumugam P.**, Genomics England, London, United Kingdom, **Bevers R.**, Genomics England, London, United Kingdom, **Bleda M.**, Genomics England, London, United Kingdom, **Boardman-Pretty F.**, Genomics England, London, United Kingdom; William Harvey Research Institute, Queen Mary University of London, London, United Kingdom, **Boustred C. R.**, Genomics England, London, United Kingdom, **Brittain H.**, Genomics England, London, United Kingdom, **Brown M. A.**, **Caulfield M. J.**, Genomics England, London, United Kingdom; William Harvey Research Institute, Queen Mary University of London, London, United Kingdom, **Chan G. C.**, Genomics England, London, United Kingdom, **Giess A.**, Genomics England, London, United Kingdom, **Griffin J. N.**, **Hamblin A.**, Genomics England, London, United Kingdom, **Henderson S.**, Genomics England, London, United Kingdom; William Harvey Research Institute, Queen Mary University of London, London, United Kingdom, **Hubbard T. J. P.**, Genomics England, London, United Kingdom, **Jackson R.**, Genomics England, London, United Kingdom, **Jones L. J.**, Genomics England, London, United Kingdom; William Harvey Research Institute, Queen Mary University of London, London, United Kingdom, **Kasperaviciute D.**, Genomics England, London, United Kingdom; William Harvey Research Institute, Queen Mary University of London, London, United Kingdom, **Kayikci M.**, Genomics England, London, United Kingdom, **Kousathanas A.**, Genomics England, London, United Kingdom, **Lahnstein L.**, Genomics England, London, United Kingdom, **Lakey A.**, **Leigh S. E. A.**, Genomics England, London, United Kingdom, **Leong I. U. S.**, Genomics England, London, United Kingdom, **Lopez F. J.**, Genomics England, London, United Kingdom, **Maleady-Crowe F.**, Genomics England, London, United Kingdom, **McEntagart M.**, Genomics England, London, United Kingdom, **Minneci F.**, Genomics England, London, United Kingdom, **Mitchell J.**, Genomics England, London, United Kingdom, **Moutsianas L.**, Genomics England, London, United Kingdom; William Harvey Research Institute, Queen Mary University of London, London, United Kingdom, **Mueller M.**, Genomics England, London, United Kingdom; William Harvey Research Institute, Queen Mary University of London, London, United Kingdom, **Murugaesu N.**, Genomics England, London, United Kingdom, **Need A. C.**, Genomics England, London, United Kingdom; William Harvey Research Institute, Queen Mary University of London, London, United Kingdom, **O‘Donovan P.**, Genomics England, London, United Kingdom, **Odhams C. A.**, Genomics England, London, United Kingdom, **Patch C.**, Genomics England, London, United Kingdom; William Harvey Research Institute, Queen Mary University of London, London, United Kingdom, **Perez-Gil D.**, Genomics England, London, United Kingdom, **Pereira M. B.**, Genomics England, London, United Kingdom, **Pullinger J.**, Genomics England, London, United Kingdom, **Rahim T.**, Genomics England, London, United Kingdom, **Rendon A.**, Genomics England, London, United Kingdom, **Rogers T.**, Genomics England, London, United Kingdom, **Savage K.**, Genomics England, London, United Kingdom, **Sawant K.**, Genomics England, London, United Kingdom, **Scott R. H.**, Genomics England, London, United Kingdom, **Siddiq A.**, Genomics England, London, United Kingdom, **Sieghart A.**, Genomics England, London, United Kingdom, **Smith S. C.**, Genomics England, London, United Kingdom, **Sosinsky A.**, Genomics England, London, United Kingdom; William Harvey Research Institute, Queen Mary University of London, London, United Kingdom, **Stuckey A.**, Genomics England, London, United Kingdom, **Tanguy M.**, Genomics England, London, United Kingdom, **Taylor Tavares A. L.**, Genomics England, London, United Kingdom, **Thomas E. R. A.**, Genomics England, London, United Kingdom; William Harvey Research Institute, Queen Mary University of London, London, United Kingdom, **Thompson S. R.**, Genomics England, London, United Kingdom, **Tucci A.**, Genomics England, London, United Kingdom; William Harvey Research Institute, Queen Mary University of London, London, United Kingdom, **Welland M. J.**, Genomics England, London, United Kingdom, **Williams E.**, Genomics England, London, United Kingdom, **Witkowska K.**, Genomics England, London, United Kingdom; William Harvey Research Institute, Queen Mary University of London, London, United Kingdom, **Wood S. M.**, Genomics England, London, United Kingdom; William Harvey Research Institute, Queen Mary University of London, London, United Kingdom, **Zarowiecki M.**, Genomics England, London, United Kingdom

## Data Availability

The data analyzed in this study is subject to the following licenses/restrictions: The anonymised phenotype and genotype data from the 100k GP analysed in this study are available to registered Genomics England Clinical Interpretation Partnership members in the Genomics England Research Environment. Information regarding how to apply for data access is available at the following URL: https://www.genomicsengland.co.uk/. All data shared in this manuscript were approved for export by Genomics England, who provide oversight to ensure patient anonymity and data security. Requests to access these datasets should be directed to https://www.genomicsengland.co.uk/.
